# Plant and animal protein intake and its association with depression, anxiety, and stress among Iranian women

**DOI:** 10.1186/s12889-023-15100-4

**Published:** 2023-01-24

**Authors:** Ali Sheikhi, Fereydoun Siassi, Abolghassem Djazayery, Bijan Guilani, Leila Azadbakht

**Affiliations:** 1 Department of Community Nutrition, School of Nutritional Sciences and Dietetics, Tehran University of Medical Sciences, P. O. Box: 1416643931, Tehran, Iran; 2 Department of Clinical Psychology, University of Tehran, Tehran, Iran; 3 Diabetes Research Center, Endocrinology and Metabolism Clinical Sciences Institute, Tehran University of Medical Sciences, Tehran, IR Iran

**Keywords:** Protein, Amino acids, Depression, Anxiety, Stress, Mental health

## Abstract

**Background:**

Mental disorders are conditions that affect the usual function of the brain, causing a huge burden on societies. The causes are often unclear, but previous research has pointed out, as is the case with many other diseases, that nutrition could have a major role in it. Amino acids, the building blocks of proteins, are the main precursor of neurotransmitters (the chemical messengers in the brain) malfunction of which is heavily associated with a wide range of brain disorders.

**Methods:**

We assumed different sources of dietary protein could have different impacts on mental well-being. Hence, we decided to collect the nutritional data (with a validated and reliable semi-quantitative food-frequency questionnaire) from a sample of 489 Iranian women and investigate the association between animal and plant protein sources and the risk of depression, anxiety, and stress. Symptoms of these mental disorders were assessed using a validated Depression, Anxiety, and Stress Scales (DASS) questionnaire with 21 items.

**Results:**

After multivariable adjustment, it was shown that women in the highest tertile of animal protein intake were more likely to show symptoms of depression (OR: 2.63; 95% CI: 1.45, 4.71; P = 0.001), anxiety (OR: 1.83; 95% CI: 1.04, 3.22; P = 0.03), and stress (OR: 3.66; 95% CI: 2.06, 6.50; p < 0.001). While no significant association was seen between plant protein and any of the studied mental disorders.

**Conclusion:**

Overall, our findings suggest that a diet high in animal protein could predispose individuals to mental illnesses.

## Introduction

A mental disorder is defined as a mental pattern in which the normal personal function of an individual is disrupted or impaired. Affecting millions of people, they account for a significant proportion of the global disease burden [1]. they consist of several complications, among which depression, anxiety, and distress are the most common. It is estimated that 4.7 and 7.3% of the global population suffer from depression and anxiety, respectively [2]. Reports from Iran suggest that about 21% of Iranian adults show depressive and anxiety symptoms [3]. Also, psychological disorders are on the rise, particularly among women, and reports suggest they are twice as likely as men to be afflicted with these complications [4]. The outcomes of psychological disorders vary from poor occupational, academic, and social status to being prone to developing some chronic severe complications, including cardiovascular disease, diabetes, and cancer [5–8]. Hence, finding the best approach to control and manage these diseases is of utmost importance. Though the exact mechanism behind mental illnesses is still not fully understood, genetic and environmental factors seem to play a critical role [9, 10]. Identifying disease-modifying risk factors could be a practical approach to preventing and managing these diseases.

Evidence reveals the role of diet in the onset, severity, and duration of mental disorders [11]. Healthy eating habits have been promoted to help prevent or even treat some of the most notorious mental disorders [12]. One of the crucial components of a healthy diet is protein. It can be provided from both plant and animal food sources. Plant sources were formerly regarded as incomplete proteins. However, new studies have revealed that prudent vegetarian diets could adequately supply all essential amino acids [13]. Yet, the absorption of which is still considered lower than that of animal sources [14]. Amino acids (mostly tryptophan, phenylalanine, and tyrosine) are suggested to have significant roles in mental health as they help build neurotransmitters [11]. Neurotransmitters, such as dopamine and serotonin, are the chemicals that allow brain cells to communicate with each other, the malfunction of which has been strongly associated with various brain disorders [15]. A study showed that brain concentration of the mentioned amino acids was significantly lower in patients showing depressive behavior compared to healthy individuals [16]. Low levels of serotonin as a result of a tryptophan-depleted diet resulted in poor memory and depressed mood [17, 18].

In the present study, we aspire to find out which source of protein, animal or plant, could result in better mental outcomes. To the best of our knowledge, no study has ever investigated this matter before. Hence, we decided to conduct this cross-sectional observational study to examine the association between different protein sources and the risk of psychiatric disorders in women.

## Method

### Study design and participants

Subjects of the present study were recruited from 10 health centers affiliated with the Tehran University of Medical Sciences. The sample size was determined employing the following formula: N= [(Z_1-α_/2)^2^ P(1-P)]/d^2^. Using P = 29, d = 4.06, and α = 0.05 [19]. Our study population consisted of healthy women who were in the age range of 20–50 years old and of Iranian descent. Women who were pregnant and lactating, premenopausal, or suffered from chronic diseases such as diabetes, cardiovascular disease, cancer, kidney or liver disorders, were diagnosed with a mental illness or took drugs affecting mental status were excluded. Overall, 489 individuals were included in the study. Written informed consent was signed by each participant. This study was confirmed by the research council of the School of Nutritional Sciences and Dietetics (number: 9611323008), TUMS.

### Dietary assessment

Dietary intake was evaluated using a validated semi-quantitative food frequency questionnaire (FFQ) containing 168 food items [20]. Participants were aided by trained dietitians in completing all forms and questionnaires. Participants were asked to report the frequency of each food during the past year on a daily, weekly, monthly, or annual basis. The animal protein category was defined as the sum of meat (beef, lamb), poultry, fish, egg, and dairy. The plant protein category consisted of whole grains, refined grains, legumes, nuts and seeds, fruits, and vegetables. The amount of each food was converted to grams using household measures. Each food item was coded, and nutrients were calculated using the NUTRITIONIST IV software for Iranian foods (version 7.0; N-Squared Computing, Salem, OR, USA).

### Dietary inflammatory index (DII)

FFQ-derived dietary data were used to calculate DII scores for all participants. Shivappa et al. [21] developed this index based on 45 food and nutrients that had been assumed to be associated with one or more of the pro-inflammatory (Interleukin-1β, Interleukin-6, Tumor Necrosis Factor-α, or CRP) or anti-inflammatory biomarkers (Interleukin-4 and Interleukin-10). Then, a score was given to each food parameter based on whether it favors the odds of inflammation by shifting towards inflammatory markers (+ 1) or reduces inflammation by doing otherwise (-1), or did not produce any significant change in the inflammatory markers (0). In the current study, we calculated DII scores based on 30 food parameters (some of the nutrients listed in the above study were not available in our database) which are as follows: energy, carbohydrate, protein, total fat, monounsaturated fat, polyunsaturated fat, saturated fat, omega-3, omega-6 fatty acids, cholesterol, fiber, thiamin, riboflavin, niacin, vitamin B6, folic acid, vitamin B12, iron, magnesium, selenium, zinc, β carotene, vitamin A, C, D, E and tea, onion, caffeine, and garlic.

### Assessment of psychological profile

The psychological profile was assessed using a questionnaire of depression, anxiety, and stress scale (DASS-21), the reliability of which was previously confirmed [22]. Each of the three DASS subscales consists of 7 questions, and the answers to which contained four options and were given a score of 0 (never applied to me) to 3 (applied to me very much or most of the time). The final score was obtained by totalizing the scores of each of the three sub-scales multiplied by two. The results were interpreted as ‘normal,’ ‘mild,’ ‘moderate,’ ‘severe,’ to ‘extremely severe’ for each subscale. However, for statistical analysis, subjects were classified into two categories normal and abnormal.

### Anthropometric measurements and physical activity

The Height was measured to the nearest 0.5 cm with a tape measure while the subjects were in a standing position, with their shoulders in a normal alignment and shoes removed. The weight was measured by a digital scale (SECA, Hamburg, Germany. With an accuracy of 0.1 kg) while the subjects were barefoot and wearing a minimum of clothes. For the waist circumference (WC), the narrowest abdominal circumference between the iliac crest and the rib cage was measured. Body mass index was calculated by dividing the weight (kg) by height squared (m^2^). The amount of physical activity was recorded and presented in metabolic equivalents × h/d (Met.h/d). Activity level was ranked into four categories (light, moderate, strong, and intense). Participants’ physical activity level was calculated as Met.h/d [23].

### General information

General information, including age, marital status, smoking status, socioeconomic status (SES), chronic diseases (diabetes, cardiovascular disease, cancer, kidney or liver disorders), family history of chronic diseases, medication and supplement use, and menopausal status was obtained. SES score was evaluated as an index of socioeconomic status regarding the family situation (being head of the family, self-care, or under supervision), frequency of travel within the country and abroad, welfare status, occupational status, the head of the family’s occupational status, education (≤ Diploma > Diploma), the head of the family’s education, and family size (≤ 4, > 4 people).

### Statistical analysis

General characteristics across tertiles of animal and plant protein intake were expressed as means ± SDs for continuous variables and numbers and percentages for categorical variables. To examine the differences across tertiles, we used ANOVA for continuous variables and a Chi-square test for categorical variables. Dietary intakes of study participants across animal and plant protein tertiles were compared using ANCOVA. All values were adjusted for energy intake. We used binary logistic regressions to estimate ORs and 95% CIs for psychological profiles across plant and animal protein tertiles in crude and multivariable-adjusted models. In these analyses, age and total energy intake, SES (low, medium, and high), marital status (married, single), physical activity, supplement use (yes/no), drug use (yes/no), family history of chronic disease (yes/no), sleep time, out of home time, body size image (normal, abnormal), were controlled in the adjusted model according to the earlier data conducted on the matter. To pin down the results solely to the effects of protein, we made a further adjustment for DII. All statistical analyses were done using the Statistical Package for Social Sciences (version 21; SPSS Inc.). P < 0.05 was considered to be statistically significant.

## Results

The general characteristics of the study population across tertiles of plant and animal proteins are shown in Table 1. The total mean and standard deviation (SD) of age, weight, BMI, and physical activity of participants was 31.82 (7.68), 64.41 (12.00), and 24.45 (4.51), 39.88 (6.76), respectively. BMI and weight have shown significant differences among tertiles of plant and animal proteins. Also, participants with the highest adherence to plant protein were more physically active than those with low adherence. The frequency and percentage of participants with depression, anxiety, and psychological distress were 175 (35.8%), 280 (57.3%), and 204 (41.7%), respectively.

**Table 1:** General characteristics of participants across the tertiles of Animal protein and Plant protein

		Plant protein			*P*- value	Animal protein			*P-* value
	Total (N = 489)	T1 ≤ 25.1 (N = 170)	T2 25.2–33.1 (N = 171)	T3 ≥ 33.2 (N = 148)		T1 ≤ 32.1 (N = 165)	T2 32.2–42.8 (N = 170)	T3 ≥ 42.9 (N = 154)	
Age (year)	31.82 ± 7.68	31.20 ± 7.95	31.97 ± 7.38	32.37 ± 7.70	0.38	31.55 ± 7.86	31.45 ± 7.67	32.51 ± 7.50	0.39
BMI (kg/m2)	24.45 ± 4.51	23.66 ± 3.94	24.35 ± 4.59	25.47 ± 4.86	0.002	23.74 ± 4.32	24.52 ± 5.99	25.12 ± 4.91	0.02
Weight (kg)	64.41 ± 12.00	62.72 ± 10.89	64.42 ± 12.25	66.33 ± 12.68	0.02	62.53 ± 11.41	64.81 ± 12.03	65.98 ± 12.37	0.03
Physical activity (Met.h/d)	39.88 ± 6.76	38.86 ± 5.90	40.67 ± 7.14	40.13 ± 7.12	0.05	39.39 ± 6.29	39.79 ± 6.58	40.51 ± 7.42	0.34
**Socioeconomic status (n (%))**					0.05				0.73
Low	42 (8.6)	22 (52.5)	12 (28.5)	8 (19)		15 (38.8)	17 (34.5)	10 (26.7)	
Medium	179 (36.6)	56 (31.3)	76 (42.5)	47 (26.2)		66 (43.1)	60 (33.0)	53 (23.9)	
High	268 (54.8)	92 (34.3)	83 (31)	93 (34.7)		84 (33.5)	93 (32.3)	91 (34.2)	
**Marital status** (**n (%))**					0.06				0.48
Single	178 (36.4)	66 (37)	70 (39.3)	42 (23.7)		69 (38.7)	59 (33.3)	50 (28)	
Married	311 (63.6)	105 (33.7)	101 (32.6)	105 (33.7)		96 (30.8)	111 (35.6)	104 (33.6)	
**Education status (n (%))**					0.01				0.96
≤Diploma	166 (33.9)	54 (32.5)	50 (30.2)	62 (37.3)		59 (35.5)	57 (34.3)	50 (30.2)	
>Diploma	323 (66.1)	122 (37.7)	118 (36.5)	83 (25.8)		109 (33.7)	112 (34.6)	102 (31.7)	
**Supplement use (n (%))**					0.24				0.59
Yes	193 (39.4)	73 (38)	72 (37.2)	48 (24.8)		66 (34.1)	65 (33.6)	62 (32.3)	
No	296 (60.6)	97 (32.7)	99 (33.4)	100 (33.9)		99 (33.4)	105 (35.4)	92 (31.2)	
**Drug Use**					0.80				0.81
Yes	41 (8.4)	16 (9.4)	14 (8.2)	11 (7.4)		16 (9.7)	14 (8.2)	11 (7.1)	
No	448 (91.6)	154 (90.6)	157 (91.8)	137 (92.6)		149 (90.3)	156 (91.8)	143 (92.9)	

Values are mean ± SD for continuous variables and number (percentage) for dichotomous variables

Using one-way ANOVA for continuous variables and Chi-square test for categorical variables

Dietary intakes of the study population among tertiles of plant and animal proteins are presented in Table 2. Participants in the highest tertile of plant protein had a higher intake of whole grains, vegetables, energy, protein, carbohydrate, total fiber, vitamin A, thiamine, vitamin C, calcium, magnesium, potassium, zinc, and iron and lower intakes of total fat and vitamin B12 compared with those in the lowest tertile. We did a further investigation to see if the tertiles overlap. Frequency analysis showed that only 5.5% of the participants were in the highest tertile of both animal and plant proteins which we assume could hardly affect the result of this study.

**Table 2:** Dietary intakes of study participants across the tertiles of Animal protein and Plant proteins

	Plant protein			*P- value	Animal protein			*P- value	
	T1 ≤ 25.1 (N = 170)	T2 25.2–33.1 (N = 171)	T3 ≥ 33.2 (N = 148)		T1 ≤ 32.1 (N = 165)	T2 32.2–42.8 (N = 170)	T3 ≥ 42.9 (N = 154)		
**Nutrient items**									
Energy (kcal/d)	1712.12 ± 28.6	2087.37 ± 27.96	2502.57 ± 37.23	< 0.001	1810.44 ± 32.62	2078.39 ± 34.52	2393.97 ± 38.33		< 0.001
Protein (g/day)	73.81 ± 1.25	73.6 ± 1.09	80.47 ± 1.36	< 0.001	65.72 ± 1.01	75.04 ± 0.94	87.59 ± 1.06		< 0.001
Carbohydrate (g/day)	276.27 ± 2.63	290.45 ± 2.29	305.95 ± 2.85	< 0.001	304.52 ± 2.42	293.06 ± 2.25	272.75 ± 2.53		0.297
Fat (g/day)	82.04 ± 1.12	76.51 ± 0.98	69.62 ± 1.22	< 0.001	75.41 ± 1.11	75.56 ± 1.02	78.17 ± 1.15		0.02
Dietary fiber (g/d)	14.08 ± 0.40	16.33 ± 0.35	20.74 ± 0.44	< 0.001	17.82 ± 0.42	17.20 ± 0.39	16.66 ± 0.43		0.003
Vitamin A (RAE/day)	1105.50 ± 626.71	1306.20 ± 687.80	1765.62 ± 1054.74	< 0.001	1085.57 ± 624.70	1471.64 ± 1009.07	1579.93 ± 758.89		< 0.001
Thiamine (mg/day)	1.39 ± 0.10	1.54 ± 0.10	1.70 ± 0.02	< 0.001	1.56 ± 0.02	1.55 ± 0.01	1.49 ± 0.02		0.169
Vitamin B6 (mg/day)	1.29 ± 0.03	1.35 ± 0.02	1.36 ± 0.03	0.24	1.34 ± 0.02	1.37 ± 0.02	1.28 ± 0.03		0.06
Vitamin B12 (µg/day)	5.14 ± 0.21	4.23 ± 0.18	4.27 ± 0.23	0.005	3.46 ± 0.19	4.66 ± 0.18	5.63 ± 0.20		< 0.001
Vitamin C (mg/day)	125.53 ± 5.75	136.11 ± 5.02	165.97 ± 6.24	< 0.001	144.73 ± 5.57	148.35 ± 5.17	132.21 ± 5.82		0.11
Calcium (mg/day)	916.55 ± 325.56	1052.96 ± 316.36	1215.43 ± 365.12	< 0.001	780.37 ± 189.51	1052.42 ± 226.32	1351.18 ± 371.28		< 0.001
Magnesium (mg/day)	218.71 ± 57.37	264.06 ± 50.47	331.35 ± 81.30	< 0.001	225.88 ± 62.52	268.18 ± 64.70	315.02 ± 80.40		< 0.001
Potassium (mg/day)	2800.91 ± 850.20	3324.98 ± 698.24	4258.31 ± 1183.62	< 0.001	2857.95 ± 873.51	3440.40 ± 953.28	4015.49 ± 1131.88		< 0.001
Zinc (mg/day)	7.35 ± 2.22	8.69 ± 1.93	11.45 ± 5.03	< 0.001	6.84 ± 1.76	8.88 ± 1.75	11.62 ± 4.91		< 0.001
Fe (mg/day)	14.92 ± 4.60	20.18 ± 5.29	32.63 ± 35.38	< 0.001	18.52 ± 10.16	21.84 ± 9.41	26.28 ± 21.14		0.004
**Food items**									
Whole Grains (g/day)	378.52 ± 4.94	440.39 ± 8.32	495.08 ± 10.35	< 0.001	451.06 ± 9.47	436.98 ± 8.78	416.89 ± 9.88		0.426
Vegetables (g/day)	240.91 ± 15.38	330.40 ± 13.43	423.51 ± 16.69	< 0.001	309.98 ± 15.33	351.08 ± 14.21	324.30 ± 16.00		0.12
Fruit (g/day)	320.98 ± 18.66	309.81 ± 16.31	369.46 ± 20.27	0.07	375.97 ± 17.66	333.59 ± 16.37	287.03 ± 18.43		0.005
Legumes and nuts (g/day)	48.31 ± 3.51	63.87 ± 3.06	98.64 ± 3.81	0.07	70.50 ± 3.57	70.31 ± 3.31	66.42 ± 3.73		0.69
Dairy (g/day)	525.84 ± 18.68	509.40 ± 16.60	457.59 ± 20.26	0.06	356.36 ± 15.45	501.10 ± 14.33	654.53 ± 16.13		< 0.001
Meats (g/day)	108.78 ± 4.10	90.84 ± 3.58	88.69 ± 4.54	0.002	66.94 ± 3.42	99.94 ± 3.17	131.53 ± 3.57		< 0.001

Except for total energy, all values are presented as mean ± SD and adjusted for energy intake

*Obtained from ANCOVA.

Participants in the top tertile of animal protein consumed more dairy, meats, energy, protein, total fat, vitamin A, vitamin B12, calcium, magnesium, potassium, zinc, and iron and consumed a lower amount of fruits and dietary fiber. Moreover, there was no significant difference in the consumption of Vitamin B6, fruits, dairy, legumes, and nuts across tertiles of plant protein and intakes of carbohydrate, Thiamine, Vitamin B6, Vitamin C, whole grains, vegetables, legumes, and nuts among animal protein tertiles.

Multivariable-adjusted odds ratio (OR) and 95% Confidence intervals (CIs) for psychological profiles across tertiles of plant and animal proteins are presented in Table 3. In the crude model, a higher score of animal protein was directly related to the risk of depression (OR = 1.82; 95% CI: 1.15, 2.90; P = 0.03), anxiety (OR = 1.70; 95% CI: 1.08, 2.67; P = 0.04), and psychological distress (OR = 2.72; 95% CI: 1.71, 4.32; P = < 0.001). After controlling for potential confounders comprising age, energy intake, physical activity, number of deliveries, socioeconomic status, supplemented use, marital status, educational level, BMI, and DII, the association between depression (OR: 2.63; 95% CI: 1.45, 4.71; P = 0.001,), anxiety (OR: 1.83; 95% CI: 1.04, 3.22; P = 0.03), and psychological distress (OR: 3.66; 95% CI: 2.06, 6.50; p < 0.001) across highest vs. lowest animal protein tertiles remained significant. However, in comparison of top to bottom tertiles of plant protein, there were no significant association between depression (OR = 0.87; 95% CI: 0.55, 1.37; P = 0.21), anxiety (OR = 1.26; 95% CI: 0.80, 1.97; P = 0.47), and psychological distress (OR = 0.88; 95% CI: 0.56, 1.39; P = 0.34) with plant protein tertiles in the crude model, and the associations remained insignificant even after adjusting for confounding factors in the fully-adjusted model.

**Table 3:** Crude and multivariable-adjusted odds ratios (95% CIs) for depression, anxiety, and stress across tertiles of plant and animal proteins

Variable		Plant protein		P _trend_^b^		Animal protein		P _trend_^c^
	T1^∞^ ≤ 25.1 (N = 170)	T2 25.2–33.1 (N = 171)	T3 ≥ 33.2 (N = 148)		T1^∞^ ≤ 32.1 (N = 165)	T2 32.2–42.8 (N = 170)	T3 ≥ 42.9 (N = 154)	
**Depression**								
Crude	1.00	0.70 (0.42, 1.15)	0.87 (0.55, 1.37)	0.21	1.00	1.34 (0.84, 2.12)	1.82 (1.15, 2.90)	0.03
Model I ^a^	1.00	0.60 (0.29, 1.05)	0.77 (0.43, 1.39)	0.10	1.00	1.44 (0.89, 2.32)	2.15 (1.27, 3.64)	0.01
Model II ^b^	1.00	0.55 (0.32, 0.95)	1.14 (0.56, 2.25)	0.84	1.00	1.70 (1.01, 2.86)	2.63 (1.45, 4.71)	0.001
**Anxiety**								
Crude	1.00	0.98 (0.63, 1.51)	1.26 (0.80, 1.97)	0.47	1.00	1.25 (0.80, 1.95)	1.70 (1.08, 2.67)	0.04
Model I ^a^	1.00	0.90 (0.56, 1.43)	1.05 (0.59, 1.86)	0.78	1.00	1.53 (0.85, 1.64)	1.95 (1.28, 2.83)	0.02
Model II ^b^	1.00	1.18 (0.70, 1.99)	1.91 (0.98, 3.72)	0.06	1.00	1.45 (0.88, 2.38)	1.83 (1.04, 3.22)	0.03
**Psychological distress**								
Crude	1.00	0.72 (0.47, 1.12)	0.88 (0.56, 1.39)	0.34	1.00	1.25 (0.81, 1.92)	2.72 (1.71, 4.32)	< 0.001
Model I ^a^	1.00	0.63 (0.39, 1.01)	0.66 (0.37, 1.18)	0.15	1.00	1.37 (0.87, 2.16)	3.02 (1.75, 5.21)	< 0.001
Model II ^b^	1.00	0.79 (0.48, 1.32)	0.99 (0.52, 1.90)	0.94	1.00	1.49 (0.92, 2.40)	3.66 (2.06, 6.50)	< 0.001

^∞^ Lowest tertile considered as reference group

^a^ Adjusted for age, energy intake, socioeconomic status, physical activity, marriage status, educational status, supplement use, drug use, and BMI.

^b^ Further adjusted for DII

^c^ Obtained from logistic regression

## Discussion

The present study suggests that poultry and dairy products are the most important contributors to animal protein intake in a representative sample of the Iranian population. While rice and legumes were the most important contributors to plant protein intake.

Our findings indicated that a higher animal protein intake is associated with an increased risk of depression, anxiety, and stress in adult women. However, there was no significant association between a high plant protein intake and the mentioned mental disorders.

Mainstream medicine views mental disorders as a result of neurochemical imbalances, for instance, depression is often viewed as a serotonin imbalance, and new anti-depressants are prescribed to target the serotonin network [24]. Another primary neurotransmitter is GABA, a lack of which has been linked to anxiety. Thus many drugs that counter anxiety do so by stimulating GABA release [25].

Nutrition can play a vital role in the pathophysiology and management of psychiatric disorders by affecting the regulation of neurotransmitters. Certain amino acids (especially tryptophan, tyrosine, and phenylalanine) found in high-quality protein sources are known to be the main precursors of these neurotransmitters [26]. It was also found that the rate of brain serotonin synthesis depends on the concentrations of tryptophan in the brain [27]. Rosier et al. revealed that a dietary intervention with low levels of phenylalanine and tyrosine would cause a rapid lowering of mood in patients who recovered from depression [28].

In this study, we found that consuming more animal protein is associated with an increased risk of psychiatric disorders. There is some evidence that could justify our findings. Tryptophan is the primary precursor of serotonin [29]. To enter the brain, a carrier protein must transport tryptophan through the blood-brain barrier. However, tryptophan is in constant competition with six other amino acids (isoleucine, leucine, phenylalanine, tyrosine, and valine) to bind to the carrier [30]. Consuming rich protein sources provides the body with the mentioned amino acids in abundance, making it more arduous for tryptophan to pass through the barrier. As a result, serotonin production might be reduced. Moreover, in a clinical trial study on 18 individuals who were divided into two groups, it was revealed that the group who consumed plant-based meals during the test had higher brain tryptophan and tyrosine levels than those who consumed meals high in animal sources [31].

Another explanation may involve the metabolism of homocysteine. Homocysteine is a byproduct of animal protein as it is converted from methionine, an amino acid found abundantly in red meat. Homocysteine’s serum level further increases if folate is not adequate in the body, which is common among women [32]. Higher homocysteine levels are strongly associated with major psychiatric disorders [33]. It is suggested that elevated homocysteine levels could cause cerebral vascular disease and neurotransmitter deficiency [34].

It should be noted that several other factors could also promote diet-induced damage to mental health, including oxidative stress, insulin resistance, and inflammation [35]. They could be the inevitable outcomes of a long-term high intake of animal products that contain nutrients such as saturated fatty acid, arachidonic acid, heme iron, and cholesterol, which are known to cause inflammation [36]. Systemic inflammation could significantly affect the brain by actively transporting cytokines through the brain and disrupting neurotransmitters’ metabolism [37]. Furthermore, excessive consumption of red meat was shown to alter gut microbiota composition [38], generating bioactive metabolites that could cause neuroinflammation through the gut-brain-axis [39]. Hence, it may not be plausible to attribute the results of the study solely to the proteins, although we did try to neutralize other nutrients’ effects by controlling for DII, which is a dietary index developed to measure the potential impact of a diet on the inflammatory status of an individual [21].

Our findings were in general agreement with previous studies investigating the matter. A meta-analysis of 8 observational studies showed that meat consumption could be associated with a slightly higher risk of depression [40]. In a cross-sectional study conducted on 892 Asian residents of the United States, a vegetarian diet which was characterized by no intake of meat, poultry, and fish was found to be inversely associated with the prevalence of depression [41]. furthermore, a cohort study conducted on 3502 participants found that the Mediterranean diet, which is rich in plant-based foods and low in red meat, had an inverse relation with depression [42]. The same conclusion was drawn in another large cohort study where the relationship between the dietary approach to hypertension (DASH) diet and depression was assessed [43]. A Japanese study found that plant protein was associated with decreased prevalence of depressive symptoms [44]. A clinical study by Beezhold et al. concluded that restricting animal-based foods such as meat, fish, and poultry improved short-term mood [45].

On the other hand, Li et al. reported that protein intake from milk and milk products was inversely associated with depressive symptoms [46]. They suggested that a-lactalbumin, a whey-derived protein that is a rich source of tryptophan, could exert beneficial effects on mood and cognition.

The present study could further expand our knowledge regarding the association of the protein with mental well-being. Still, some limitations should be noted. First, the recall bias in reporting dietary intake has probably affected the results. The cross-sectional nature of our study was another limitation, as it prevented us from inferring causality. The study was performed only on females aged 20–50 years, which affects the generalizability to the larger population. Also, due to the different influence of gonadal steroids on mood [47], we could have gotten better insight into the variable of sex if men had also been present. Furthermore, it has been reported that the menstrual cycle could affect depressive symptoms, which were not regarded in our study [48]. Also, the DASS-21 is a self-reported scale based on a dimensional rather than a categorical conception of mental disorder. This scale is used to measure the severity of symptoms of anxiety, stress, and depression and is helpful for screening, not for diagnosis.

In conclusion, we found that high adherence to animal protein is associated with an elevated risk of psychiatric disorders. Future longitudinal studies are required to further our understanding of the effect of different protein sources on mental health.

## Data Availability

The data supporting this study’s findings are available upon reasonable request and with permission of Dr. Leila Azadbakht. However, restrictions apply to the availability of these data as they contain the personal information of the participants. So.

## Abbreviations

DASS-21: Depression, Anxiety and Stress Scale
BCAA: Branched-Chain amino acids
BMI: Body Mass Index
FFQ: Food frequency questionnaire
WC: Wait Circumference
CVD: Cardiovascular Disease
SFA: Saturated Fatty Acids
HDL: High-density Lipoprotein
LDL: Low-density Lipoproteins
OR: Odds Ratio
CI: Confidence interval
